# Parasitic Zoonoses: One Health Surveillance in Northern Saskatchewan

**DOI:** 10.1371/journal.pntd.0002141

**Published:** 2013-03-21

**Authors:** Janna M. Schurer, Momar Ndao, Stuart Skinner, James Irvine, Stacey A. Elmore, Tasha Epp, Emily J. Jenkins

**Affiliations:** 1 University of Saskatchewan, Saskatoon, Saskatchewan, Canada; 2 National Reference Centre for Parasitology, Research Institute of the McGill University Health Center, Montreal General Hospital, Montreal, Quebec, Canada; 3 Division of Infectious Diseases, Royal University Hospital, Saskatoon, Saskatchewan, Canada; 4 Population Health Unit, La Ronge, Saskatchewan, Canada; The George Washington University Medical Center, United States of America

## Abstract

We report the results of a joint human-animal health investigation in a Dene community in northern Saskatchewan, where residents harvest wildlife (including moose, bear, elk, and fish), live in close contact with free roaming dogs, and lack access to permanent veterinary services. Fecal analysis of owned and free-roaming dogs over two consecutive years (N = 92, 103) identified several parasites of public health concern, including *Toxocara canis, Diphyllobothrium* spp., *Echinococcus/Taenia*, *Cryptosporidium* spp. and *Giardia* spp. Administration of pyrantel pamoate to a subset of dogs (N = 122) in the community in the first year was followed by reduced shedding of *T. canis* and other roundworms in the second year, demonstrating the potential utility of canine de-worming as a public health intervention. Using direct agglutination tests with confirmatory indirect fluorescent antibody test, 21% of 47 dogs were sero-positive for exposure to *Toxoplasma gondii*. Using enzyme-linked immunosorbent assay (ELISA) sero-prevalence rates in 201 human volunteers were as follows: *Toxoplasma gondii* (14%), *Echinococcus granulosus* (48%), *Toxocara canis* (13%) and *Trichinella* spp. (16%). Overall 65% of participants were sero-positive for at least one parasite. A survey administered to volunteers indicated few associations between widely accepted risk factors for parasite exposure and serological status, emphasizing the importance of environmental transmission of these parasites through soil, food, and waterborne routes.

## Author Summary

Parasites are ubiquitous, and while some parasitize only one host, others are capable of crossing species barriers. Zoonotic parasites move between animals and people, and in some cases cause significant veterinary, medical and/or public health problems. Such parasites may be more prevalent in areas where veterinary and medical services are scarce, and especially if sanitation infrastructure is suboptimal. Additional risk factors include reliance on country foods, proximity to pets that come in contact with wildlife, and eating undercooked or raw fish and game. We visited one northern Indigenous community over two consecutive years to determine the prevalence of internal parasites in dogs, as well as to demonstrate the effect of selective deworming on reducing environmental contamination by zoonotic parasites. In addition, we collected blood samples and administered surveys to human volunteers in order to explore the relationship between exposure to four zoonotic parasites and several widely accepted risk factors for exposure (e.g. pet ownership). Our findings indicate that levels of parasite exposure in this community were higher than similar studies conducted in other Canadian Indigenous communities. Public health interventions that utilize a one health strategy by integrating medical, veterinary and environmental expertise may be the most effective approach in reducing human and animal exposure to parasites in this community.

## Introduction

Northern Indigenous peoples have recently been identified as being at high risk for acquiring parasitic zoonoses due to socioeconomic factors and a close relationship with the land [1]. Hunting and fishing are common activities in northern Saskatchewan where consumption of country foods is an integral part of a traditional Dene diet and a very important contribution to food security in regions where commercial foods are often expensive, unavailable, and nutritionally inadequate [2]. Free-roaming dogs continue to play important roles in Indigenous communities as wildlife deterrents, security, companion animals, and occasionally transport [3]. Human exposure to zoonotic parasites might be above average in these communities if free-roaming dogs have access to raw game or fish and subsequently shed infective stages of parasites in areas frequented by people. Other risk factors for exposure to zoonotic parasites include contaminated or inadequately treated drinking water, handling and consumption of locally caught and inadequately cooked game or fish, challenges of waste disposal in remote environments, and/or absence of veterinary services [4]–[6].

Recently, the prevalence of intestinal parasitic infection in dogs was reported to be as high as 71% in northern Saskatchewan [7]. Several genera of zoonotic parasites have been identified in canine populations including *Echinococcus/Taenia, Giardia, Cryptosporidium, Toxocara* and *Diphyllobothrium*, for which dogs may serve as sources or sentinels for human exposure [4], [7]. Few studies have simultaneously sampled people as well as free-roaming dog populations to determine their role as sources or sentinels for human infection with parasitic zoonoses [8]. A number of human sero-prevalence studies have been conducted in northern and predominantly Indigenous regions of Canada; however, none of these have focused on Dene communities in northwestern Canada, which share many of the same socioeconomic and public health concerns as Inuit in Nunavut, and Inuit and Cree in Nunavik and the James Bay region of northern Quebec [9]–[16]. Zoonotic infectious such as echinococcosis and trichinellosis occur more frequently in northern and Indigenous populations; however, incidence rates of other zoonotic parasites are currently unknown for northern Saskatchewan [17].

We conducted research relating to veterinary public health in one Indigenous community in the Keewatin Yatthé (KY) health authority over a two year period (2010, 2011). The KY region is located in the northwestern part of Saskatchewan, and is one of three public health regions that encompass northern Saskatchewan [9]. Approximately 10 600 people reside in this area, of which 94% self-identify as Indigenous (primarily Métis, Dene and Cree); a proportion similar to that seen in James Bay and Nunavik, Quebec. Social determinants of health significantly contribute to health inequities in this population, and include the high cost of food, housing shortages, low income, and high unemployment. This health region has a shorter life expectancy, higher all-cause mortality, and higher rates of chronic and communicable disease (including diarrheal outbreaks, tuberculosis, hepatitis C and HIV/AIDS) than the provincial average [9].

In this paper we study canine endoparasitism and human exposure to four parasites of medical concern: *Echinococcus granulosus, Trichinella, Toxocara canis* and *Toxoplasma gondi*. Social and behavioural risk factors for exposure to these parasites are also explored.

## Materials and Methods

### Participants

In 2011, we visited one community in northern Saskatchewan with an approximate population of 2400 people and, primarily through word of mouth, recruited 201 volunteers over the age of 4 years (female N = 77; male N = 124). In addition, we sampled dog feces collected from the ground and samples from client-owned dogs brought to a veterinary service clinic in the community in 2010 and 2011.

### Human serology and risk factor assessment

Approximately 5 mL of whole blood was collected directly into serum-separator tubes (BD; Franklin Lakes, NJ) and kept refrigerated. Tubes were centrifuged at 3000 rpm for 10 minutes within 8 hours of collection, and sera transferred to snap-top mini centrifuge tubes. Serum samples were sent to the National Reference Centre for Parasitology (McGill University, Montreal, QC) and tested for IgG antibodies against *T. gondii* (Diagnostic Automation/Cortez Diagnostics, Inc, Calabasas, CA), *Trichinella* spp., *Toxocara canis* and *E. granulosus* by using an in-house developed IgG and IVD Research (Carlsbad, CA) enzyme-linked immunosorbent assay (ELISA). Criteria for interpretation of serology results are provided in Table 1. Equivocal results were designated as sero-negative. Each participant was also asked to respond to a survey pertaining to risk factors for parasite exposure. Questions addressed pet ownership, feeding practices, barriers to veterinary care, hunting, fishing and personal consumption of country foods. Not all participants completed the surveys to entirety, and some small children were grouped under their parents' surveys.

**Table 1:** Results of serological analyses and criteria for sero-status in people.

Parasite	Measurement	Criteria and this study's results		
		Negative	Equivocal	Positive
*Toxocara canis*	Optical Density	<0.25	≥0.25 to <0.35	≥0.35
Number Samples		164/201	10/201	27/201
*Trichinella*	Optical Density	<0.25	≥0.25 to <0.35	≥0.35
Number Samples		149/201	19/201	33/201
*Echinococcus granulosus*	Optical Density	<0.35	≥0.35 to <0.45	≥0.45
Number Samples		77/201	27/201	97/201
*Toxoplasma gondii*	Units IgG (IU/mL)	<1	NA	≥1
Number Samples		173/201	-	28/201

### Canine fecal surveillance

Approximately 300–400 dogs are estimated to reside in this community. We conducted canine fecal collection and analysis in this community during the month of June over two consecutive years (2010: N = 92; 2011: N = 103) to test the effectiveness of anthelmintic administration as a public health intervention. Fecal samples were obtained by rectal collection of client-owned dogs brought to a mobile veterinary service clinic (2010: N = 31; 2011: N = 34), as well as by ground collection throughout the community (2010: N = 61; 2011: N = 69) as a measure of environmental contamination. All dogs (N = 122) brought to the mobile clinic in 2010 were treated with pyrantel pamoate as per label dose, and owners were given additional medication along with instructions to repeat the treatment after 7–10 days. The ratio of male to female dogs brought to the clinic was approximately one to one, and all intact animals were desexed. Approximately half of the clinic animals were within one year of age.

For ground collected feces around the community, collection of fresh fecal samples was prioritized, with older samples (grey or white) being rejected. Samples were stored in sealed plastic bags and kept in coolers with ice during the collection period (1–2 days). Feces were transported to the University of Saskatchewan (Saskatoon, SK) and stored at −80 degrees Celsius for five days to inactivate taeniid eggs. A quantitative sucrose centrifugation flotation was used to quantify and identify parasite eggs and cysts from approximately 5 grams wet weight of feces (modified from [18]). *Giardia* spp. cysts and *Cryptosporidium* spp. oocysts were identified using a sucrose gradient flotation and a commercial immunofluorescent assay (Waterborne Inc.; New Orleans, LA) on approximately 1 gram wet weight of feces [19].

### 

#### Canine serology

We conducted sero-surveillance of *Toxoplasma gondii* for dogs brought to the mobile veterinary service clinic in this community in 2011 (N = 47). Approximately 3 mL of whole blood was collected directly into serum-separator tubes (BD; Franklin Lakes, NJ), and chilled on ice. Sera were collected as described for the human study. Sera were analyzed for the presence of antibodies to *T. gondii* at the University of Saskatchewan (Saskatoon, SK) using a modified direct agglutination test (Biomerieux Toxo-Screen DA kit; Montreal, QC) at a 1∶40 dilution. Samples with equivocal results on this test were confirmed using an indirect fluorescent antibody test (IFAT; VMRD, Pullman, WA).

### Ethics

All participants provided written informed consent and those under the age of 18 provided written consent from a parent or guardian to participate. Individual serology results were mailed back to the participant and/or their primary care physician. The human study was reviewed and approved by the University of Saskatchewan Biomedical Research Ethics Board (REB 11-07), as well as by the Keewatin Yatthé Health Region and the community leader. The animal fecal and serology studies were reviewed and approved by the University of Saskatchewan Animal Research Ethics Board (2009-0126 and 2010-0159, respectively), which adheres to the Canadian Council on Animal Care (CCAC) standards. Dog owners provided consent for their animals to be sampled, while consent for ground collection of dog feces was provided by the community leader.

### Statistical methods

Human serology and survey data were entered into a spreadsheet and analysed using logistic regression to identify associations between outcomes (sero-status) and risk factors (SPSS, Chicago, Illinois, USA). The strength of association between an outcome and variables was reported as an odds ratio (OR) with 95% confidence intervals (CI) (OpenEpi version 2.3.1, Atlanta, GA, USA). Risk factors were tested for statistical significance in a multivariate model using manual backward elimination. Risk factors were considered confounders if their inclusion or exclusion changed the effect estimate of another risk factor by more than 10%. In the case of correlated risk factors, only one was included in the final model. A chi-square test was used to determine if proportions were significantly different (p-value<0.05).

## Results

### Human serology and risk factor assessment

Of the 77 women and 124 men (N = 201) sampled in the Keewatin Yatthé public health region, 65% had been exposed to at least one of four zoonotic parasites (Table 2). The participation rate was approximately 8%, however a number of potential volunteers were turned away due to limited phlebotomy supplies. The prevalence of diagnostically relevant titres was as follows: *Echinococcus granulosus* 47.8% (96/201), *Toxocara canis* 13.4% (27/201), *Trichinella* 16.4% (33/201) and *Toxoplasma gondii* 13.9% (28/201). Of those who were sero-positive, 24% had been exposed to 2 parasites, and 8% had been exposed to 3; no person had been exposed to all 4 zoonoses. Co-exposure occurred most commonly between *E. granulosus* and *Trichinella* (19/201; 9.5%), with similar proportions between *E. granulosus* and the remaining parasites: *T. canis* (17/201; 8.5%) and *T. gondii* (14/201; 7%).

**Table 2:** Sero-surveillance for *Echinococcus granulosus, Trichinella, Toxocara canis* and *Toxoplasma gondii* in northern Indigenous regions (Canada) [10]–[16].

Reference	Location	Sample Size (N)	*Toxoplasma gondii*	*Echinococcus granulosus*	*Toxocara canis*	*Trichinella* spp
			Sero-prevalence (%)			
Campagna, 2011	James Bay, QC	250	5	4	3	1
Sampasa-Kanyinga, 2012	James Bay, QC	267	9	0.7	4	0
Egeland, 2010	Inuvialuit, NT	362	6	0.7	0.7	0.7
Egeland, 2010	Nunatsiavut, NU	310	8	0.4	1	1
Messier, 2012	Nunavik, QC	917	60	8	4	1
Tanner, 1987	Northern QC	1195	30	2	10	2
Levesque, 2007	Mistissini, QC	50	10	0	4	0
Schurer, 2012	Northern SK	201	14	48	13	16

NB: All studies used the same tests by the same laboratory except Tanner 1987.

Analysis of the survey identified several practices that could potentially expose people to zoonotic parasites (Table 3). Nearly all participants ate locally acquired foods including meat, fish, mushrooms and berries. Popular methods of wild game and fish preparation included drying, smoking or cooking; while raw foods were rarely consumed. Of pet owners, 74% fed raw meat and 70% fed fish to pets on a regular basis. Participants aged 5–17 had higher odds of exposure to *T. canis* (OR 3.4 95% CI 1.2-10) than those over the age of seventeen; and feeding pets non-commercial dog food increased the odds of exposure by 15 times (95% CI 1.8-126). Increased odds of exposure to *T. gondii* were observed in participants older than fifty (OR 9.4 95% CI 1.1-77) and those who did not own pets (OR 3.8 95% CI 1.3-11.3), however gender and hunting/trapping are probable confounders for pet ownership.

**Table 3:** Potential risk factors for exposure to four zoonotic parasites in a northwestern Saskatchewan community.

Risk Factor		Odds Ratios (95% Confidence Interval)			
	Sample Size (N)	*Toxoplasma gondii*	*Echinococcus granulosus*	*Toxocara canis*	*Trichinella*
Gender (male)	201	2.2 (0.9–5.3)	1.2 (0.7–2.1)	1.9 (0.8–4.8)	1.5 (0.7–3.4)
Does not hunt/trap	188	1.5 (0.7–3.6)	**1.9 (1.1–3.4)**	1.2 (0.5–2.8)	0.5 (0.3–1.2)
Wild game consumption	196	0.5 (0–4.9)	0.3 (0–2.9)	0.5 (0–4.5)	0.2 (0–1.4)
Does not own a pet	199	**3.8 (1.3–11.3)**	1.2 (0.7–2.1)	1.3 (0.5–3.1)	1.8 (0.8–4.2)
Non-commercial pet diet	73	0.4 (0.4–3.8)	1.9 (0.7–5.0)	**15 (1.8–126)**	1.0 (0.2–4.0)
Age 5–17*	174	0.2 (0–2)	1.8 (0.7–4.6)	**3.4 (1.2–10)**	2.0 (0.7–5.8)
Age over 50**	68	**9.4 (1.1–77)**	0.3 (0.1–0.8)	0.4 (0.1–1.3)	0.4 (0.1–1.5)

* compared with all other ages.

** compared with 5–17 age group.

### Canine feces and serum

Examination of canine feces identified five parasite genera of relevant zoonotic potential in this community, including *Diphyllobothrium, Toxocara, Echinococcus/Taenia*, *Cryptosporidium* and *Giardia*. Ground collected fecal samples were observed to have more parasites (2010: 51% 31/61: 2011: 35%, 24/69) than fecal samples of dogs brought to the clinic (2010: 48%, 13/31; 15%, 5/34). Chi-squared analysis indicates that the decrease in overall prevalence of endoparasitism from 2010 (48%; 42/92) to 2011 (28%; 29/103) is statistically significant (p-value 0.005) (Table 4). During this time period overall decreases were noted in roundworms (*Toxocara* 9%, *Toxascaris* 5%, *Uncinaria* 11%); while the prevalence of tapeworms increased (Taeniid 4%, *Diphyllobothrium* 13%). Examination of client-owned dogs in this region in 2011 demonstrated an exposure prevalence of 21% (10/47) to *T. gondii*.

**Table 4:** Prevalence of canine intestinal parasites identified through quantitative sucrose flotation and immunofluorescent assay.

	Prevalence (%)		Intensity Mean, Median, Minimum-Maximum (eggs per gram)	
Community ID	KY-2010	KY-2011	KY-2010	KY-2011
*Toxocara*	8/92 (9%)	0/103 (0%)	77, 70, 10-230	0
*Toxascaris*	10/92 (11%)	6/103 (6%)	2316, 31, 3-22500	1652, 64, 5-9660
*Uncinaria*	10/92 (11%)	0/103(0%)	174, 34, 3-1005	0
Taeniid	0/92 (0%)	4/103 (4%)	0	124, 123, 3-248
*Diphyllobothrium*	2/92 (2%)	16/103 (15%)	586, 586, 8-1165	1795, 23, 3-15000
*Isospora*	1/91 (1%)	0/103 (0%)	^*^470, 470	0
*Giardia*	11/89 (12%)	2/95 (2%)	185, 100, 33-733	183, 183, 33-333
*Cryptosporidium*	14/98 (14%)	4/95 (4%)	83, 50, 33-200	417, 250, 133-1033
^*^Overall	42/92 (48%)	29/103 (28%)		

* Overall prevalence was calculated as the number of samples with at least one parasite type divided by the total sample number.

## Discussion

This study shows that the prevalence of exposure to zoonotic parasites for residents of northwestern Saskatchewan is higher than previously reported in other Canadian sero-prevalence studies. As well, dogs residing in this area appear to encounter and be infected by potentially zoonotic parasites at higher levels than dogs residing in Saskatoon (a provincial urban centre) [20]. Exposure to *T. canis, T. gondii* and possibly *E. granulosus* was observed in both people and dogs, indicating that dogs may act as sources and sentinels for human infections. Wild meat consumption, pet ownership and hunting/trapping are generally considered to increase the risk of exposure to zoonotic parasites, however our analysis indicated that there might be a slight overall protective effect. This demonstrates the complexity of parasite transmission routes and the possibility of protective immunity and/or traditional knowledge regarding harvesting and preparation of wild foods.

*Echinococcus granulosus* is a cyclophyllid cestode with a worldwide distribution, causing serious veterinary, medical and economic concerns for highly endemic regions [21]. Human infection with *E. granulosus* causes hydatid disease, or echinococcocis, which is generally characterized as the formation of larval cysts in the liver and lungs. The average annual incidence rate of hydatid disease in Canada is 0.72 cases per million people, and is higher in women than men (RR 1.92, 95% CI 1.29-2.87) and north of the 55^th^ parallel (RR 4.88, 95% CI 2.52-9.44) [17]. Hospital records in both Canada and the United States show Indigenous people to be at higher risk of infection [22], [23]. In another recent study conducted in a Saskatchewan Indigenous community, 11% of 103 people were sero-positive for *E. granulosus*, and at least two cases of hydatid disease were identified [8; S. Skinner, unpubl. data). The sero-prevalence of 48% to *E. granulosus* in the current study is substantially higher than the 0–4% reported in other Indigenous communities of similar northern latitude, analysed using the same test and by the same laboratory, the National Reference Centre for Parasitology [10]–[16]. We are not aware of any clinical cases in this community at the current time; however, there is no formal surveillance for this parasite in Canada. There is a strong possibility that the unexpected level of exposure is due to cross-reactions with other helminths. Diphyllobothriasis cases are relatively common in this region [J. Irvine, unpubl. data], and other possibilities include the liver fluke, *Metorchis conjunctus*, and various *Taenia* species. We know that *Diphyllobothrium* is present in dogs in this community (Table 4), and *Metorchis* has historically been reported in dogs, wolves and people in SK [5], [24]–[26].

*Trichinella* nematodes have long been associated with consumption of undercooked pork; however, livestock production practices have virtually removed this parasite from the domestic Canadian swine herd [27], [28]. North American wildlife may be infected with one of five zoonotic genotypes of *Trichinella*, and consumption of these animals has been the primary cause of Canadian trichinellosis outbreaks since the 1970s [27]–[32]. In northern Saskatchewan, exposure is most commonly attributed to the consumption of wild bear meat; while in Inuit regions of Nunavut and Nunavik, exposure is associated with consumption of marine mammals such as walrus [30]–[31], [33]–[36]. The national annual incidence rate of trichinellosis is only 0.09 cases per million people, however rates are significantly higher in Nunavut and Nunavik (42 cases per million people) [17]. In Canadian northern and Indigenous communities the sero-prevalence for *Trichinella* in people ranges between 0–5.5%, which is far lower than our reported exposure prevalence of 16.4%. Antibodies to this parasite can persist up to 19 years, making it difficult to detect recent changes in exposure frequency [37].

*Toxocara canis* is an ascarid nematode that cycles primarily among canids, and commonly infects domestic dogs in Canada and around the world. People may become exposed through accidental ingestion of eggs shed in dog feces, or by ingestion of tissue cysts in the undercooked meat of paratenic hosts. Toxocariasis, characterized by visceral or ocular larval migrans, is not commonly reported in Canada, but may cause serious health effects. In our study youth were more likely to be exposed than adults, consistent with observations that children are at highest risk for infection when they play in sand or soil contaminated by dog feces, or due to pica [38]–[40]. We found that dog ownership was not a risk factor for exposure to *T. canis*, similar to one other study in Canada [41], thus supporting the importance of environmental (versus direct) transmission of this parasite. Feeding non-commercial diets to family dogs significantly increased the odds of human exposure to *T. canis*. This may be due to increased transmission to dogs via the paratenic host route, followed by human contact with eggs shed in dog feces. Alternatively, feeding non-commercial pet diets may correlate with other variables, such as poverty and occupational exposures to soil, that put people at risk of exposure [42]–[43]. Sero-prevalence for *Toxocara* was between 0.7–4% in recent studies in Inuit and Cree communities in northern Canada [11]–[16]. Our reported prevalence of 13.4% is therefore much higher than that observed in Canadian communities north of the 60^th^ parallel, consistent with observations of restricted survival of *T. canis* eggs at colder temperatures [38], [44]–[46]. It is on par with the 13.9% reported in the general population of the United States between 1988 and 1994, although this was dominated by samples from the southern USA where this parasite may have increased levels of transmission [42]. Reducing risk of exposure to this parasite could focus on regular deworming of dogs, timely disposal of feces (the eggs are not immediately infective), and preventing dogs from defecating in areas where children play.

*Toxoplasma gondii* has a global distribution, and is one of the most important parasites in the Canadian North [1]. This protozoan has a complex lifecycle involving felids as definitive hosts and a wide variety of vertebrate species as intermediate hosts. In our study population, routes for dog exposure include feeding raw meat to dogs, ingestion of garbage and wildlife. As well, sero-positive status in dogs is associated with age, diet, hospitalization, and health status; a sample of young, stray dogs had the lowest level of sero-positivity [47]–[48]. We observed a lower level of exposure to *T. gondii* in our population (21%) than dogs tested in Alberta, the Northwest Territories and Ontario (33–63%) [4], [48], which may be due to the relatively young population sampled.

Dogs are not known to spread *T. gondii* to people, however, our finding suggest that people in the community may be at risk due to share exposure routes. People become infected by ingesting or handling raw meat, ingesting contaminated drinking water, handling infective cat feces, or by congenital transmission, blood transfusion or organ transplant [49]. We report a sero-prevalence of 13.9%, which is comparable to the NHANES estimate of 10.8% in the United States [50], and generally higher than that reported elsewhere in Canada using the same test in the last 6 years (5–10%). Inuit in Nunavik, Quebec have one of the highest sero-prevalences reported (30–60%), and are thought to have a unique constellation of risk factors (Table 2). Risk factors include gender (female>male), drinking water sources, regular disinfection of water reservoirs, and limited education [10]–[16],[51]. Exposure to *T. gondii* in the Keewatin Yatthé region was statistically higher with age (>50 years), and with those who did not own a pet; however, confounding variables might nullify the effect of pet ownership on sero-status.

Saskatchewan currently has the highest incidence rate of Human Immunodeficiency Virus (HIV) in Canada, at double the national average. Indigenous patients are disproportionally affected, and represented 79% of HIV/AIDS cases in 2009 [52], [53]. HIV/AIDS is a serious risk factor for development of clinical toxoplasmosis. Mortality attributed to toxoplasmosis in AIDS cases in Europe and the United States is estimated to be 30% and 10%, respectively [49], [54]. The higher proportion of immune-compromised individuals in northern Saskatchewan combined with limited veterinary services, frequent contact with wildlife, and lifting of previously restrictive climate conditions, may lead to emergence of previously uncommon zoonotic pathogens as public health concerns (*T. gondii* and *Cryptosporidium*).

The prevalence of endoparasitism in client-owned dogs from this community was similar to levels previously found in remote areas of Saskatchewan [5], [55]. Ground-collected fecal samples did not represent the true parasite prevalence in this community as multiple samples may have originated from the same animal. However this method is an effective tool for estimating the overall level of environmental contamination as well as for identifying local parasites of zoonotic concern; in this case *T. canis*, Taeniid, *Diphyllobothrium, Giardia*, and *Cryptosporidium*. The voluntary nature of human and canine recruitment was another limitation of this study; however, we considered this strategy as crucial in building trust with the community. The purpose of blood testing was not revealed during recruitment, and only 17% of participants were aware that pathogens could move between animals and people. Thus, people with concerns of parasite exposure were not more likely to participate. Sampling of client-owned animals was biased towards pets with owners who considered veterinary services important. However, we considered this effect minimal because all dog owners permitted blood and/or fecal collection, all veterinary services were cost-free, and local volunteers rounded up stray dogs. Shedding of roundworm eggs (*T. canis, Toxascaris* and *Uncinaria*) decreased in 2011, following administration of pyrantel pamoate to dogs brought to the mobile veterinary service unit in 2010. This could reflect drug effectiveness, decreased transplacental and transmammary transmission of *T. canis* due to spaying female dogs, and/or the effect of having fewer puppies, which are the primary source of environmental contamination. Alternately, the observed concomitant decreases in protozoa, which are not affected by pyrantel pamoate, suggest that changes in parasite prevalence may result from factors such as annual climate variations and altered animal husbandry practices. Whatever the cause, the overall decrease of parasitism in dogs bought to the clinic and in environmental contamination is a benefit to public health; however, the increased prevalence of cestode eggs demonstrates the additional need for cestocidal treatment to reduce risks to human health. Finally, this study reinforces that surveillance and management of zoonoses in remote areas requires a One Health approach incorporating both veterinary and public health interventions, tailored to concerns at the local level.
